# Low Incidence of *Clostridioides difficile* Infection in Patients Receiving Outpatient Parenteral Antibiotic Therapy at an Urban Academic Medical Center

**DOI:** 10.1093/ofid/ofaf314

**Published:** 2025-05-27

**Authors:** Christopher Vo, Jared Coe, Tabeer Ahmed, Kelsie Cowman, Yi Guo, Priya Nori

**Affiliations:** Division of Infectious Diseases, Department of Medicine, Montefiore Medical Center, Bronx, New York, USA; Division of Infectious Diseases, Department of Medicine, Montefiore Medical Center, Bronx, New York, USA; Division of Infectious Diseases, Department of Medicine, Montefiore Medical Center, Bronx, New York, USA; Division of Infectious Diseases, Department of Medicine, Montefiore Medical Center, Bronx, New York, USA; Division of Infectious Diseases, Department of Medicine, Montefiore Medical Center, Bronx, New York, USA; Division of Infectious Diseases, Department of Medicine, Montefiore Medical Center, Bronx, New York, USA

**Keywords:** *Clostridioides difficile* infections, CDI, hospital-acquired infections, nosocomial infections, outpatient parenteral antibiotic therapy

## Abstract

Data on *Clostridioides difficile* infection (CDI) among patients receiving outpatient parenteral antibiotic therapy (OPAT) are limited. Herein, we describe characteristics of OPAT patients with CDI at a large academic medical center. Despite prolonged antibiotic exposure, the incidence rate of CDI was low.

The Centers for Disease Control and Prevention's Emerging Infections Program reported 116.1 cases of *Clostridioides difficile* infection (CDI) per 100 000 persons in 2022, with 56% of CDI cases having antibiotic exposure in the prior 12 weeks [1]. CDI is the most common healthcare-associated infection in the United States [2]. The epidemiology of and risk factors for developing inpatient CDI are well established and increasing rates of CDI are also observed in nonacute settings [3–6]. Prolonged antibiotic exposure is strongly associated with CDI development [3–5, 7–9]. Outpatient parenteral antibiotic therapy (OPAT) programs manage patients on prolonged parenteral or oral antibiotics following hospital discharge, and the contribution of OPAT to the incidence of CDI is an emerging area of study [10, 11]. Prior studies report a CDI incidence of less than 1% among OPAT patients [10–12]. In this study, we describe clinical characteristics and outcomes of OPAT patients who developed CDI at our medical center.

## METHODS

### Study Design and Participants

This is an observational retrospective study conducted at Montefiore Medical Center, a 3-hospital, >1400-bed academic institution in the Bronx, New York. It included all adult patients discharged on OPAT between 1 January 2019 and 31 December 2023. Antimicrobial regimens were recommended by inpatient infectious diseases consultants per Montefiore OPAT enrollment criteria. OPAT enrollment was defined as referral to the OPAT program once an inpatient or outpatient infectious diseases consultant determined the need for long-term oral or parenteral antibiotics *≥*2 weeks post–hospital discharge or as a direct outpatient OPAT start.

Our study was approved by the Albert Einstein College of Medicine Institutional Review Board (IRB# 2016–6338). We included patients >18 years old enrolled in OPAT in the study timeframe. Patients who developed CDI within 6 months after OPAT enrollment were categorized as CDI attributable to OPAT therapy. CDI was defined as having diarrhea (watery stools >3 times/day) and a positive stool *C difficile* test per the hospital algorithm at the time (initial glutamate dehydrogenase/toxin test [Techlab, Inc], then confirmatory polymerase chain reaction [Cepheid GeneXpert] test if discordant glutamate dehydrogenase and toxin results). Patients were excluded if they had CDI diagnoses <1 year prior to OPAT enrollment. A 1-year cutoff was utilized to decrease confounding from recent CDI given the higher than baseline risk of recurrence, and to maximize OPAT as the exposure of interest. Patients were also excluded if they developed CDI during the index hospitalization prior to discharge into OPAT care.

The following data were collected via electronic health records: antibiotic regimen and duration (≥14 days, 15–30 days, 31–42 days, >42 days), medical comorbidities, demographics (age, sex, etc), OPAT diagnosis (eg, endocarditis, bone and joint infection), and other clinical factors. Previously validated definitions for CDI with high-risk features/immunocompromise were utilized [1, 3–9, 13–17]. Proton pump inhibitor (PPI) use was measured as any PPI prescription or orders between OPAT enrollment date and CDI test order date. The primary outcome was the percentage of confirmed CDI cases after OPAT enrollment (number of CDIs/total enrolled OPATs in same timeframe). Secondary outcomes for CDI cohort patients included all-cause mortality, antibiotic class, subsequent hospitalization, and number of CDI risk factors.

### Statistical Analysis

We summarized patient characteristics and outcomes using the appropriate descriptive statistics, frequencies and percentages, and means and interquartile ranges. The CDI incidence rate within 6 months of OPAT enrollment was assessed per 1000 patient-days. Patient-days were calculated as days from OPAT enrollment to CDI or 180 days for patients who did not have CDI.

## RESULTS

Between 1 January 2019 and 31 December 2023, there were 3134 patients enrolled in OPAT, with a total of 4338 OPAT episodes managed by the Montefiore OPAT program, with multiple patients managed repeatedly for new infection episodes during the study timeframe. There were 286 (6.6%) direct OPAT enrollments from the outpatient setting, with the remainder enrolled from an inpatient setting. No patients with multiple OPAT episodes had CDI more than once. All demographic and clinical information pertaining to this cohort are summarized within Table 1.

**Table 1.** Demographic and Clinical Factors of Patients With *Clostridioides difficile* Infection Receiving Outpatient Parenteral Antibiotic Therapy (n = 54)

Demographics	Count	(%)
Age, y, median (IQR)	62.5	(52–70)
Sex		
Male	26	(48.1)
Female	28	(51.9)
Race/Ethnicity		
Hispanic	16	(29.6)
Non-Hispanic Asian	2	(3.7)
Non-Hispanic Black	25	(46.3)
Non-Hispanic White	6	(11.1)
Other	2	(3.7)
Unknown	3	(5.6)
Clinical factors		
OPAT diagnosis		
Bone/joint	27	(50.0)
Endocarditis	1	(1.9)
Intra-abdominal infection	7	(13.0)
Skin and soft tissue infection	3	(5.6)
>1 OPAT diagnosis	1	(1.9)
Other	15	(27.8)
Bacteremia	8	(55.3)
CNS infection	2	(13.3)
Complicated UTI	1	(6.7)
Empyema	1	(6.7)
Endovascular	2	(13.3)
Fungal	1	(6.7)
CDI risk factors		
Chronic kidney disease comorbidity	27	(50.0)
Immunocompromised	24	(44.4)
Age >65 y	22	(40.7)
Proton pump inhibitor use	19	(35.2)
Recent hospital stay in past 90 d	50	(92.6)
Recent nursing home stay in past 90 d	20	(37)
OPAT use		
Duration, d, median (IQR)	42	(28–42)
Received multiple antibiotic classes	19	(35.2)
Antibiotic class received		
Antifungal	3	(5.6)
Carbapenem	3	(5.6)
Cephalosporin	30	(55.6)
1st generation	9	(30)
2nd generation	0	(0)
3rd generation	11	(36.7)
4th generation	10	(33.3)
5th generation	0	(0)
Clindamycin	0	(0)
Glycopeptides	10	(18.5)
Linezolid	1	(1.9)
Metronidazole	6	(11.1)
Penicillin	12	(22.2)
Quinolone	6	(11.1)
Rifampin	1	(1.9)
Tetracycline	3	(5.6)
Trimethoprim-sulfamethoxazole	1	(1.9)
Outcomes		
Mortality within 90 d of CDI date	6	(11.1)
Subsequent admission after OPAT enrollment	35	(64.8)

Data are presented as No. (%) unless otherwise indicated.

Abbreviations: CDI, *Clostridioides difficile* infection; CNS, central nervous system; IQR, interquartile range; OPAT, outpatient parenteral antimicrobial therapy; UTI, urinary tract infection.

A total of 54 patients met CDI inclusion criteria, with a prevalence of 1.2% and CDI incidence rate of 0.096 per 1000 patient-days (within 6 months of OPAT enrollment). There were 26 (48.1%) male and 28 (51.9%) female patients. CDI patients were predominantly non-Hispanic Black (46.3%) or Hispanic (29.6%), reflecting the overall borough demographics [18]. The median number of CDI risk factors was 3. The most common risk factors were recent hospitalization within 90 days (n = 50), chronic kidney disease (n = 27), immunocompromised status (n = 24), age >65 years (n = 22), and recent nursing facility stay within 90 days (n = 20). Nineteen (35.2%) patients had PPI use prior to CDI. The most common OPAT diagnoses were bone/joint infection (50%); other conditions including central nervous system infections, bacteremia, or urinary tract infection (27.8%); intra-abdominal infection (13.0%); skin and soft tissue infection (5.6%); and endocarditis (1.9%).

Thirty-five patients (64.8%) required readmission for any cause. Of these, 19 (54.3%) came from home, and 16 (45.7%) from other post–acute care settings. Among those requiring readmission, 3 patients (8.6%) were diagnosed with CDI as outpatients prior to readmission, 21 (60%) were diagnosed within the first 7 days of admission, and 11 (31.4%) were diagnosed after >7 days. Only 1 patient (1.9%) in the CDI cohort was enrolled into OPAT from the clinic setting.

Among patients who developed CDI, the median duration of antibiotic therapy was 42 days (range, 14 to ≥180 days). The most common antibiotic classes were cephalosporins (n = 30 [55.6%]), penicillins (n = 12 [22.2%]), and glycopeptides (n = 10 [18.5%]). Three patients (5.6%) received antifungals with antibiotics. Eighteen patients (33.3%) received multiple antibiotic classes. Nineteen (35.2%) patients’ regimens included anaerobic coverage (eg, metronidazole, clindamycin, penicillin/β-lactamase inhibitor combinations, carbapenems, second-generation cephalosporins, and quinolones). Mortality and readmission rates among all Montefiore OPAT patients between 1 January 2019 and 31 December 2023 were 8.4% and 15.8%, respectively. Among the CDI group, mortality and readmission rate within 90 days of positive CDI test were 11.1% (n = 6) and 64.8%, respectively.

## DISCUSSION

In this single-center study of OPAT patients at an urban academic medical center with predominantly non-White patients, CDI was uncommon (prevalence of ∼1.2% of patients and incidence rate of 0.096 per 1000 patient-days). While Wong et al reported a higher incidence rate of 0.26 cases/1000 patient-days, overall, studies confirm that CDI is rare in OPAT patients [10–12].

Aside from antimicrobial exposure, hospitalization in the preceding 90 days, immunocompromise, and age (>65 years) were the most frequent risk factors, consistent with prior literature [3–9, 13, 14]. Many patients had recent admissions to skilled nursing facilities. Like prior literature, antibiotic classes prescribed more frequently in CDI patients were cephalosporins (n = 30), penicillins (n = 12), and glycopeptides (n = 10) [4, 7–9, 17]. Most patients (60.7% [n = 34]) developed CDI when receiving >30 days of total antibiotics (including inpatient and outpatient days), supporting the established relationship between duration of antibiotic exposure and CDI [7–9, 17]. Mortality and readmission rates among all Montefiore OPAT patients were 8.4% and 15.8%, respectively, as compared to the 11.1% (within 90 days of CDI diagnosis) and 64.8%, respectively, in CDI patients, reflecting poorer outcomes in this group. This observation prompts consideration for additional prevention strategies [2, 5].

CDI is a multifactorial process influenced by age, prolonged antibiotics, severity of principal illness, length of stay, and host gut microbiome, etc [2–9, 13–17]. Despite prolonged and broad-spectrum antibiotic exposures, the low prevalence of CDI in our cohort suggests potential protective characteristics of treatment outside the acute care setting. Of note, there were 286 (6.6%) direct outpatient enrollments into OPAT during the study timeframe, but only 1 of 54 (1.9%) CDI patients belonged to the direct outpatient enrollment group. OPAT patients may have a restoration of previous gut and environmental bacterial flora following hospital discharge and a cessation of acid-suppressing medications. However, further exploration of ecological differences is needed [2–6, 12, 16, 17, 19–21]. Additionally, our findings highlight the impact of significant infection control and diagnostic stewardship challenges to CDI prevention in acute care.

Our study has notable limitations, including the use of retrospective and observational data from a single medical center without a comparator group of OPAT patients without CDI. CDI diagnoses occurring outside our medical center were not captured, possibly undercounting CDI cases. However, patients were likely to present within our 11-facility health system for any adverse consequences of OPAT due to close communication between patients, the OPAT team, infusion staff, or nursing facility staff. We did not compare our cohort to hospitalized patients receiving a similar duration or types of antibiotics who also developed CDI or conduct gut microbiome studies in hospitalized versus OPAT patients. Although we did compare readmissions and 90-day mortality between the CDI-OPAT cohort and the overall OPAT cohort and noted worse outcomes due to CDI, further investigation and comparison of OPAT patients who did and did not develop CDI is a planned area of future study.

## CONCLUSIONS

CDI was uncommon in our OPAT population despite receipt of prolonged antibiotics and multiple comorbidities. Efficient hospital discharge or avoidance of hospitalization of patients requiring prolonged antibiotics from the acute care setting is a priority. Home and skilled nursing-based antibiotic therapies offer several advantages, including a reduced incidence of CDI. Further study is needed to directly compare clinical characteristics of OPAT patients who did and did not develop CDI and differences in gut microbiota between hospitalized versus nonhospitalized patients with prolonged antibiotic exposure.

## Notes

***Patient consent.*** This is a retrospective study using de-identified data and was approved by the Institutional Review Board of Montefiore Medical Center. This study did not include any factors necessitating patient consent.
